# Fast-track program of elective joint replacement in hip and knee—patients’ experiences of the clinical pathway and care process

**DOI:** 10.1186/s13018-019-1232-8

**Published:** 2019-06-21

**Authors:** Urban Berg, Marie Berg, Ola Rolfson, Annette Erichsen-Andersson

**Affiliations:** 1 Department of Orthopaedics, Institute of Clinical Sciences, Sahlgrenska Academy, University of Gothenburg, Gothenburg, Sweden; 2 Department of Surgery and Orthopaedics, Kungälv Hospital, Kungälv, Sweden; 3 Institute of Health and Care Sciences, Sahlgrenska Academy, University of Gothenburg, Gothenburg, Sweden; 4 Swedish Hip Arthroplasty Register, Gothenburg, Sweden

**Keywords:** Total hip arthroplasty, Total knee arthroplasty, Fast-track, Clinical pathway, Care process, Quality of care, Person-centered care

## Abstract

**Background:**

The clinical pathway and care program in elective total hip and knee replacement (THR/TKR) has, during the last decade, undergone considerable changes in many countries influenced by the concept of fast-track surgery, resulting in a very short hospital stay. Studies into patients’ experiences of the entire fast-track program, from decision-making regarding surgery until recovery 3 months after surgery, are lacking. The aim of the study was to increase the knowledge about patients’ experiences of the clinical pathway and care in a fast-track program of elective THR/TKR in order to identify factors that may influence recovery and clinical outcome.

**Methods:**

A qualitative research design was chosen with data collected from interviews 3 months after surgery and analyzed using an inductive content analysis method. In total, 24 patients from three hospitals with a fast-track care program were included in the study: 14 women and 10 men, 13 with THR and 11 with TKR. The mean age was 65 years (range 44–85).

**Results:**

The analysis identified three chronological phases in the clinical pathway: preparation, hospital stay for surgery, and recovery. In the preparation phase, patients’ experiences and involvement in the planning of the operation were highlighted. The need to know the risks and expectations of recovery and outcome were also central, although there was great diversity in needs for information and involvement. In the hospital stay for the surgery phase, there were mainly positive experiences regarding admission, early mobilization, and early discharge. Experiences about the recovery phase focused on management of daily life, rehabilitation program, and recovery. Rehabilitation involved uncertainty as to whether or not the progress was normal. The recovery phase was also filled with questions about unfulfilled expectations. Regardless of the different phases, we found the importance of a person-centered care to be a pervasive theme.

**Conclusion:**

Our study supports the view that a person-centered approach, from surgery decision until recovery, is an important element in optimizing care in a THR and TKR fast-track care program. More focus on the period after hospital discharge may improve recovery, patient satisfaction, and functional outcome.

## Background

Joint replacement in hip and knee is effective in reducing pain and improving function and quality of life for patients with osteoarthritis or other joint destruction [1–3]. In Sweden, more than 30,000 total joint replacements in hip (THR) and knee (TKR) are performed annually with an average age at surgery of about 68 years, almost 60% of whom are women [4, 5]. Even if the results of THR and TKR are good with low frequencies of reoperation and revision, the percentage of dissatisfied patients is between 10 and 30% according to studies from different countries [6–8]. In particular, patients undergoing TKR report unfulfilled expectations regarding knee function, pain relief, and quality of life [9].

The clinical pathway and care programs in elective total hip and knee replacement are quite complex processes [10–13]. They should ensure the correct indication of surgery, optimize the patient preoperatively, and minimize the risks, but also facilitate the recovery resulting in a good functional outcome and high patient satisfaction. During the last decade, the clinical pathway and care programs in elective joint replacement have undergone considerable changes in many countries influenced by the concept of fast-track surgery [10, 14]. Fast-track aims to reduce physiological and psychological stress related to surgery with the aim of enhancing early mobilization and rapid recovery [15]. Care programs based on the fast-track principles focus on evidence-based methods related to the surgical procedure and post-discharge function [16, 17], include multiprofessional collaboration in all phases, and have resulted in very short hospital stay [10, 18]. Limited attention has been paid to the patients’ experiences.

Preoperative patient information and education is a cornerstone in hip and knee replacement care programs, especially in a fast-track program [10], although it is unsure whether it offers benefits over usual care in terms of reducing anxiety or improving postoperative outcomes with respect to pain, function, health-related quality of life, and adverse events [19]. However, a more recent study has identified that adequate patient information is a crucial factor for early hospital discharge and management of daily life at home after arthroplasty in a fast-track program [20], while another study has validated the importance of multimodal patient education tailored to individual preferences and experiences [21].

Effective pain treatment is an important issue in the fast-track program of lower limb arthroplasties that influence early hospital discharge and a fast recovery period at home [20, 22]. However, it has also been pointed out that it may be difficult for the nursing staff to adapt to variations and meet individual needs of the patients within a standardized care process [23]. Some studies focus on the discharge procedure and patients’ experiences after hospital discharge. Especially in elderly patients, the early discharge may be stressful with regard to managing daily life and rehabilitation [24].

Other areas in the care process of special interest from the patients’ perspective are “experiences of healthcare” and “involvement and understanding in care decisions.” Patients in the UK interviewed early after THR and TKR surgery described fear of the unknown and that they did not know enough to make informed choice [25]. But it has also been concluded that the patients’ experience of healthcare could be enhanced by further attention being paid to concepts of patient-centered care [26].

Although there is an increasing knowledge about important factors in the care process from the patients’ perspective and how it may influence the recovery after THR and TKR, most studies focus on the hospital stay and early recovery phase. There is a lack of studies regarding patients’ experiences from surgery decision until recovery 3 months after surgery. The aim of this study was to explore the patients’ perspective and experiences of undergoing THR and TKR surgery within the entire fast-track care process**.**

## Method

### Design

As the aim of the study was to increase knowledge about patients’ experiences, a qualitative research design was chosen with data collected from interviews and analyzed using an inductive content analysis according to Elo and Kyngäs [27].

### Participants and recruitment

In order to obtain variation and saturation of informative data responding to the research question, we chose a strategic sample of patients from three different hospitals in the western region of Sweden who underwent total hip (THR) and total knee (TKR) replacement operations, of both sexes and different ages. All three hospitals used a fast-track care program. The exclusion criteria were inability to communicate in the Swedish language or cognitive dysfunction. Almost 60 patients received information about the interview study. At two hospitals, the patients were invited and recruited in conjunction with stitch removal 3 weeks after surgery. At one hospital, the patients received the information and invitation to participate in the interview study upon discharge from the hospital. The information was given by staff members who were not involved in the study. In total, 29 patients gave written consent to participate, but five were excluded in order to avoid too many patients from one hospital. Thus, 24 patients were included in the study: eight from a university hospital (A), and seven and nine respectively from two district hospitals (B and C); 14 women and 10 men, 13 with THR and 11 with TKR. The mean age was 65 years (range 44–85), and all had been operated in a fast-track care program. Two patients with THR and two with TKR had previously been operated on the contralateral side. For details, see Table 1.

**Table 1:** List of interviews and patient data

Interview number	Age	Sex	Joint	Hospital	Length of hospital stay (LOS, days)	Interview timing (days after surgery)
1	56	Female	Hip	B	1	87
2	68	Male	Knee	C	1	82
3	77	Male	Knee	C	1	90
4	80	Female	Hip	C	1	90
5	70	Male	Knee	C	1	91
6	85	Female	Hip	C	2	90
7	68	Female	Hip	C	1	90
8	64	Female	Hip	C	1	93
9	75	Male	Hip	C	1	85
10	64	Female	Hip	B	1	95
11	80	Female	Hip	B	1	92
12	62	Female	Knee	B	2	92
13	63	Female	Knee	B	1	91
14	71	Male	Knee	B	1	111
15	59	Male	Hip	C	1	83
16	44	Female	Hip	A	2	93
17	53	Female	Hip	A	1	94
18	50	Male	Knee	A	1	74
19	53	Female	Knee	A	1	97
20	57	Male	Knee	A	1	98
21	73	Female	Hip	A	2	89
22	68	Female	Hip	A	1	96
23	64	Male	Knee	A	3	83
24	65	Male	Knee	B	1	86

*A* university hospital, *B* district hospital, *C* district hospital

### Data collection

The patients were contacted by telephone by one of the researchers (UB), and an interview was planned 3 months after surgery. The interviews were arranged at a mutually convenient time and place and carried out from May to November 2017. Most patients preferred to be interviewed at home. The interviews were semi-structured and started with open questions such as “Tell me about your experiences from the care of your hip/knee operation.” The patients were encouraged to speak freely. During the interviews, the interviewer asked for clarification such as “What do you mean?”, “Can you describe more?”, and “Can you give an example?” Supplementary open questions were used when necessary in order to cover all phases of the clinical pathway, from surgery decision until the actual situation 3 months after discharge. Special attention was paid to issues pointed out as important by the participants. The interviews, lasted on average 50 min (range 33–74), were audiotaped and transcribed verbatim.

### Data analysis

The first step was to gain familiarization of the content by reading all interviews 2–3 times (UB). Some interviews were read by two other researchers (AEA and MB). Next, the text was read again to select analysis units containing informative data. The selected units were confirmed by AEA and UB continued with coding, grouping, and categorization in collaboration with AEA and MB. Subcategories were formed and organized in generic categories according to the different phases of the clinical pathway (UB, AEA, MB).

## Result

The analysis identified three chronological phases in the clinical fast-track pathway: preparation, hospital stay for surgery, and recovery. Preparation deals with the planning and preparation of the patient from the operation decision until the admission at hospital. This phase is mostly 3 months but may be longer. Hospital stay for surgery includes the period from arrival at the hospital until discharge 1–3 days after the surgical intervention. Recovery, the third phase, starts after the discharge from the hospital and comprises rehabilitation, regaining of function, and return to normal daily life. In all three phases, subcategories were identified, which were sorted in generic categories. An overview of the result is given in Table 2. For structural reasons, the categories have numbers but these do not indicate any ranking.

**Table 2:** Summary of patients’ experiences in the care of total hip and knee replacement

Clinical pathway	Generic categories	Subcategories
Phase 1: preparation—from surgery decision until hospital admission	1.1 Confirmation that surgery is needed	1.1.1 Fear of not being accepted for surgery 1.1.2 Satisfaction when decision was made 1.1.3 Importance of shared decision-making
	1.2 Planning the date of surgery	1.2.1 Frustration when not knowing the date, and satisfaction when knowing it 1.2.2 Desire to influence the timing of surgery 1.2.3 Fear that the operation may be canceled
	1.3 Planning the anesthesia	1.3.1 Fear of being awake and having unpleasant experiences during surgery 1.3.2 Fear of complications of spinal anesthesia 1.3.3 Importance of shared decision-making
	1.4 Information about care and outcome of surgery	1.4.1 Diversity in information needs 1.4.2 Scanty information about the recovery 1.4.3 Influenced by information from other sources
Phase 2: hospital stay for surgery	2.1 Admission on the day of surgery	2.1.1 Recognition and a feeling of familiarity 2.1.2 Affirmation and seen by the staff
	2.2 Early mobilization after surgery	2.2.1 Mentally prepared and safe to be mobilized 2.2.2 Hesitation but ready to cooperate
	2.3 Early discharge	2.3.1 Acceptance and satisfaction 2.3.2 Objections and worries
Phase 3: recovery—after discharge from hospital	3.1 Managing daily life	3.1.1 Safety when having support at home 3.1.2 Diversity in pain control
	3.2 Rehab program and recovery	3.2.1 Different needs for personal coaching 3.2.2 Uncertainty about progress
	3.3 Feedback and follow-up	3.3.1 Concerns about unfulfilled expectations 3.3.2 Need for further explanations from the surgeon

Here follows a description of the formulated subcategories, sorted by phase. Quotations are from the interviewees identified as I 1 to I 24.

## Preparation phase

### Confirmation that surgery is needed

At the initial outpatient visit (meeting the orthopedic surgeon for clinical assessment and to discuss eligibility for joint replacement), patients feared that they would not be accepted for surgery due to not fulfilling the criteria of severe pain and functional limitations, or being considered too young. This fear was strengthened by scaring information from friends and other sources: “I was terrified. Because just that day I didn’t have so much pain. I thought, he won’t believe me” (I 16); “I was a bit scared since I’ve always heard in any event that if one is too young to be operated on, then they don’t do so. Like that. That was really what I was most afraid of” (I 18).

Once the surgery decision was made, there was a feeling of relief and satisfaction.

“Once it was clear... that I would be operated on, that was the most important thing” (I 12); “I was just glad that I would have the operation. And I wasn’t particularly nervous either” (I 18). Involvement in decision-making regarding surgical treatment increased the active involvement of the patient. “I participated and decided when I wanted the operation. … I felt that I was participating more when it was time to do it, and it was unavoidable” (I 13).

### Planning the date of surgery

It was very important to know when the operation would be carried out. It was frustrating to be in suspense waiting without knowing how long time it would take to get a date for surgery.

“I thought that it was taking far too long time. But unfortunately, it dragged on and on. Then I thought, I can’t agree to this” (I 3); “So I waited and waited and got no feedback, and the only question I had was ‘What’s my status?’ but it was difficult to contact the unit” (I 23).

However, getting a planned date for surgery gave a feeling of satisfaction, safety, happiness, and excitement. But sometimes there was also a fear of cancelation due to disturbances in the hospital planning or new health problems such as infections and fresh skin lesions, which could inhibit the operation. “The heart pounds, when the letter comes it pounds enormously … because you don’t know what the date will be. And when you see the date, you’re so happy … you’ve finally got a date, that you can mentally begin to focus on something.” (I 17); “I was terrified of that, that I would have a sore or I would have a cold, since then everything would have been postponed. So I did not take the bus or anything during the final week and tried to stay away from crowds as much as possible” (I 21). Some patients had a desire to influence the timing of surgery. “My employer was nagging me and wondered when I would have the operation, in order to plan for a substitute” (I 13).

### Planning the anesthesia

In the preoperative planning of the anesthesia, the patients met the anesthesiologists. Many patients stated that they preferred general anesthesia or at least being sure that they would not be awake during surgery**.** More precisely, there was a fear of hearing unpleasant noises or being aware of what was going on. “I said that I didn’t agree to being awake … as much anesthetic as possible, I said” (I 2); “I don’t want to hear them sawing and hammering and things like that. I don’t want that” (I 6).

There was also a fear that something could go wrong with the spinal anesthesia, that there would be a permanent loss of sensibility or motricity. “You’re afraid that it will go wrong … that perhaps you will not regain the feeling or … Even if you are aware that it does not happen so often, but even so, there’s nevertheless some worry”(I 1).

Spinal anesthesia was proposed to be used as the standard method of anesthesia. Despite this, there was a dialog between the patient and the anesthesiologist as to whether general or spinal anesthesia should be used. The patient’s preference was accepted if it seemed reasonable. After listening to the arguments, the patient could also be convinced to accept the suggestion from the anesthesiologist. “I’d rather get general anesthesia since I’ve been operated on many times and have never had problems being anesthetised … And then he said ‘Yes, in that case we’ll do so’. Very friendly. That’s how it was” (I 11).

### Information about care and outcome of surgery

Most patients were satisfied with the information about the surgery, although some patients needed to know more, while others did not want to receive detailed information about risks and how the surgery would be performed. “I’m that kind of person, so that if they hadn’t given me I would have forced them to give me … I want to be prepared for what they’re going to do … I want to know about the details.” (I 17); “In fact I want to know as little as possible about the procedure. No, I’m not really so fond of these kinds of operations” (I 13).

The information about the postoperative rehabilitation and recovery was perceived as scanty: “I would have liked to know more about the period after the surgery. No one told me about that. Maybe I had needed to be more prepared because it was very hard, at least the first 3-4 weeks” (I 13).

It was common for patients to obtain information from friends, relatives, and the Internet. This information was sometimes misleading. Some patients had heard that an artificial joint lasts only 10 years, and after that, you will be sitting in a wheelchair. Others had been told the recovery was very quick and easy, while others had been told that the pain after the operation is unbearable. “Before the operation I’d heard that it was so horrible to operate the knee, it was the worst operation one could have, it involved pain and everything” (I 24).

## Hospital stay for surgery

### Admission on the day of surgery

There were almost no objections to admission in the early morning on the day of surgery. The preoperative visit some weeks before the operation had prepared the patients. When arriving at the hospital, the patients experienced recognition and a feeling of familiarity. “I thought that everything was so well prepared. Everything flowed so well. I felt safe. The aspect of meeting people beforehand. … I’d been there previously. I think the whole arrangement was excellent” (I 4). Furthermore, upon admission, the patients felt that they were seen and affirmed by the staff and that the care was focused on them. “Everyone kept an eye on me, I was visible. No one lost me” (I 12); “Everyone was super-friendly and they really focused on me” (I 21).

### Early mobilization after surgery

Patients described that they were mentally prepared and felt safe to get up from the bed assisted by the staff and start mobilization within a few hours after the operation. “I knew that everyone has to do so. Up as quickly as possible and all that.” (I 7); “I received information that... after the operation you end up in the recovery room and that there one should get up and stand directly after … Yes, I’ll probably manage that” (I 19).

However, some patients had doubts as to whether it could be possible to be mobilized immediately after the operation. They hesitated but agreed to cooperate and were surprised that they were able to stand and walk so early after surgery. “It was very strange. I thought, will this be painful? Can I rely on it, what can happen? Lots of thoughts go through your head. But it’s just a question of trying, and it worked very well” (I 8).

### Early discharge

Most patients accepted discharge the day after surgery without objection, since they had received clear and concise information about the intended length of hospital stay in the preparation phase. Some even expressed satisfaction at leaving the hospital so early. “I was convinced it would be just one night, if everything was okay. That’s what I’d read in the information brochure, that it’s usually … Yes. But it nevertheless felt good … I could cope thanks to my painkillers, so I thought nevertheless that it’s good to come home” (I 1).

Not all patients were satisfied with a very short hospital stay. They expressed objections and worries, despite having received information about the criteria for discharge. Patients not fulfilling the discharge criteria stayed one or two extra nights. “Yes, with such an operation, I’m shocked that people are sent home so early, because obviously one is worried and perhaps feels more secure … Perhaps being in two, three nights in order to grasp what’s happened and to obtain information and care” (I 17).

## Recovery phase

### Managing daily life after discharge

At the beginning of the recovery phase it was of great importance to have support from a partner, relatives or friends to manage daily life at home. “I think it’s important to have help in the home … one needs help with shopping and preparing food and so forth … and you are not allowed to drive a car” (I 10). Pain control was another issue of importance in daily life after discharge from the hospital. There was great diversity in the experience of pain. The amount of medication needed against the pain, and its duration, varied a lot between patients regardless of whether the hip or knee joint was replaced, but expressions of severe pain were more common in patients operated with TKR. “I needed more, I never had enough. I didn’t want to take an overdose either. That was the thing that felt hopeless and disconsolate... that I didn’t experience any relief so that I could relax and feel hope” (I 12); “I’ve hardly had any pain, I’ve really only taken the tablets that I was forced to take” (I 15).

### Rehab program and recovery

Personal coaching by a physiotherapist was, for some patients, crucial in order to be motivated and to know how to perform the exercises in the rehab program. However, some patients preferred to continue the rehab program themselves without the involvement of a physiotherapist. “I was at his place today, in the morning, and I train there. And he helps me and checks and gets me to do a few other exercises and so … a bit more personal coaching” (I 18). There was sometimes uncertainty about the progress of mobility, walking ability, and level of physical activity in the rehabilitation and recovery. Was the progress normal or not compared to the average patient and expectations from the professionals? “Sometimes when I’m depressed, I think that there’s something wrong with me, I think that things are improving so slowly” (I 7); “perhaps one needs to have small goals... so that you see that things are going in the right direction, am I too slow or too fast” (I 20).

### Feedback and follow-up

Some patients conveyed concerns about unfulfilled expectations at the time of interview 3 months after surgery. The absence of, or scanty about, a follow-up program by the orthopedic surgeon was a source of unanswered questions. “In any event it concerns how one goes and what one can expect. Limping like this. Yes, I think it’s a bit strange that the doctor didn’t try to get information about how the operation had gone in greater detail, about how the patient is feeling after the operation” (I 10). Even if the early period of recovery seemed normal, patients expressed a need for more explanations about the operation, the expected outcome, and the future. “I’d have nothing against being allowed to come in and discuss it.” (I 11); “I think that perhaps it’s a little strange to have hardly spoken with the doctor afterwards... it wasn’t more than two minutes the day after.” (I 15).

## Discussion

The present study explores the patients’ experiences in the routine care of patients from three different hospitals, all of them with a fast-track program without patient selection. It adds valuable knowledge about the patients’ perspective of the care process and what issues are of special importance. In the preparation phase, i.e., from surgery decision until hospital admission, our findings concern information and patient involvement. Our findings echo other studies. Patients wanted to know if surgery is needed, when and how the operation should be performed, but also information about the risks and expectations of recovery and outcome. For some patients, the timing of surgery was important in order to plan sick leave from work and to arrange support at home after discharge from the hospital. In the fast-track concept, structured oral and written information is a key [10, 20]. Some patients were satisfied just to know that they were scheduled to be operated and left to the professionals the decision of when and how the operation should be done, as well as the choice of surgeon and rehab program. Other patients had an enormous need for information and wanted to know as much as possible about the preparation, the operation, the care, and the rehabilitation in order to feel safe. The first meeting with the orthopedic surgeon was of special importance and may have influenced the experience of the following steps in the care process.

In the second phase, the hospital stay for surgery, the patients’ narratives expressed mainly positive experiences. Even if some patients found the hospital stay too short due to early discharge, overall, there was general satisfaction with care, with a feeling of safety and being affirmed and seen by the staff. Our findings are similar to a study of arthroplasty patients based on questionnaire with free text responses, which concluded that positive patient experiences were closely linked to effective patient-health professional interactions and logistics of the hospital processes [26].

The recovery phase was the most insufficient and weak part of the fast-track program. This comprised scanty information about the recovery and rehabilitation progress. There was uncertainty as to what was normal or not, unfulfilled expectations remained, and patients needed support. This finding corresponds to a study on fast-track care of total hip and knee replacement in Norway [28]. It emphasizes that there is a need for more individualized and adapted information prior to discharge and greater multidisciplinary follow-up in order to improve pain management and rehabilitation during the first 6 weeks after surgery. New methods using web-based programs for individualized feedback may be an option in the future. Obviously, the discharge procedure and the early recovery period are a challenge, but according to our study, there is still a need for follow-up after 3 months or later due to unanswered questions and unfulfilled expectations related to recovery and outcome.

Regardless of the different phases, the findings indicate that person-centered care, including active patient involvement and partnership, is important for the patients from the surgery decision until recovery. These aspects of care are in congruence with essential parts of person-centered care as described by Ekman et al. [29]. To be seen and affirmed by the staff was one of the positive experiences at the admission and hospital stay. The diversity of informational needs and diversity as regards pain control were subcategories that highlighted the importance of accepting the patient as a subject with personal needs. Involvement in decision-making upgraded the patient to a partner, who participated more actively in the preparation, care, and rehabilitation [30]. When continuity was assured, it gave a feeling of satisfaction and safety that enabled a partnership*.*

Our study revealed the importance of a person-centered approach in the entire care process as a complement to the standardized care program. The acceptance of the patient as a partner actively involved in all phases may be a key to further improving the care process.

### Strengths and limitations

It is a strength that we had a strategic sample of patients from both sexes and different ages undergoing either THR or TKR. This ensured a variation of patient narratives and increased the transferability and external validity [31], thus reflecting diverse experiences in a fast-track program of elective total joint replacement. Another strength is that the entire care process was included, from surgery decision to the recovery phase. This increases the understanding of how the different phases influence the patients. A limitation is that it is difficult to explore in depth every part of the care process with one interview, which has to cover a period of more than 6 months with a multitude of experiences.

## Conclusion

Patients’ experience of elective total hip and knee replacement in fast-track programs adds valuable knowledge of important factors in the care process. Our study indicates that more focus on the period after hospital discharge may improve recovery, patient satisfaction, and functional outcome. The findings highlight that a person-centered approach is important for patients in a fast-track care program of THR and TKR, from surgery decision until recovery. Further studies are warranted, to explore how and if a person-centered approach can optimize care and be an integral part of standardized fast-track programs.

## Data Availability

The datasets analyzed during the current study are not publicly available due to confidentiality reasons, but are available from the corresponding author on reasonable request.

## Abbreviations

THR: Total hip replacement
TKR: Total knee replacement
